# Exercise alleviates cardiac remodelling in diabetic cardiomyopathy via the miR-486a-5p-Mst1 pathway

**DOI:** 10.22038/IJBMS.2020.50808.11564

**Published:** 2021-02

**Authors:** Dong Sun, Haichang Wang, Yanhui Su, Jie Lin, Mingming Zhang, Wanrong Man, Xinglong Song, Liang Zhang, Baolin Guo, Kaikai Hao, Dongdong Sun

**Affiliations:** 1 Department of Cardiology, Tangdu Hospital, Air Force Medical University of PLA, Xi’an 710038, P.R. China; 2 Department of Cardiology, Xijing Hospital, Air Force Medical University of PLA, Xi’an 710032, P.R. China; 3 Heart Hospital, Xi’an International Medical Center, Xi’an 710100, P.R. China; 4 Department of Comprehensive Surgery of The Second Medical Center, General Hospital of Chinese People’s Liberation Army, Beijing 100853, P.R. China

**Keywords:** Apoptosis, Diabetic cardiomyopathy, Exercise, Mst1 (STK4), miR-486a-5p

## Abstract

**Objective(s):**

Physical exercise has emerged as an effective therapy to mitigate cardiac remodelling in diabetic cardiomyopathy (DCM). The results of our previous studies revealed mammalian sterile 20-like kinase 1 (Mst1) is a key regulator of the progression of DCM. However, the precise molecular mechanism of physical exercise-induced cardiac protection and its association with Mst1 inhibition remain unclear.

**Materials and Methods:**

Wildtype and Mst1 transgenic mice were challenged with streptozotocin (STZ) to induce experimental diabetes and were divided into sedentary and exercise groups. The DCM phenotype was evaluated by echocardiography, Masson’s trichrome staining, TUNEL and immunoblotting analyses. The exercise-regulated miRNAs targeting Mst1 were predicted by bioinformatic analysis and later confirmed by RT-qPCR, immunoblotting, and dual-luciferase reporter assays. In addition, cultured neonatal mouse cardiomyocytes were subjected to simulate diabetes to elucidate the underlying mechanisms.

**Results:**

Compared to the sedentary diabetic control, physical exercise inhibited Mst1 and alleviated cardiac remodelling in mice with DCM, as evidenced by decreases in the left ventricular end-systolic internal dimension (LVESD) and left ventricular end-diastolic internal dimension (LVEDD), increases in the left ventricular ejection fraction (LVEF) and left ventricular fractional shortening (LVFS), attenuation of collagen deposition, and the suppression of apoptosis. Bioinformatic analysis and apoptosis assessments revealed exercise exerted protective effects against DCM through miR-486a-5p release. Moreover, luciferase reporter assays confirmed miR-486a-5p directly suppressed the expression of Mst1, thereby inhibiting the apoptosis of cardiomyocytes subjected to high glucose treatment.

**Conclusion:**

Physical exercise inhibits cardiac remodelling in DCM, and the mechanism is associated with miR-486a-5p release-induced Mst1 inhibition.

## Introduction

The prevalence of diabetes has been increasing, and the burden of diabetes should be of great concern (1, 2). Cardiovascular complications are lethal consequences of diabetes, accounting for approximately 80% of diabetes-induced deaths (2). In particular, diabetic cardiomyopathy (DCM), which shows the characteristics of impaired left ventricular diastolic and later systolic dysfunctions, cardiac hypertrophy, myocardial fibrosis and myocyte cell death, leads to a higher risk of heart failure and mortality independent of traditional risk factors such as hypertension, coronary heart disease, and valvular heart disease (3-6). With the increasing prevalence of diabetes and the effective control of the other common causes of heart failure, diabetes-related cardiomyopathy and heart failure may become increasingly prominent. Therefore, research on the pathophysiology, treatment and prevention of DCM is of increasing significance in the clinical settings. Currently, treatments for DCM consist of glycaemic control and anti-heart failure therapies, all of which are nonspecific and have relatively unsatisfactory outcomes. A number of novel therapies, such as the use of anti-oxidants (7), dietary medium-chain fatty acids (8), phosphatidylinositol 3-kinase inhibitors (9), polyunsaturated fatty acids (10-12) and visceral adipose tissue-derived serine protease inhibitors (13) have been shown to be cardioprotective. However, some of the results of these studies need further verification, and additional studies are needed for the precise control of DCM. 

Mammalian sterile 20-like kinase 1 [Mst1; also known as serine/threonine-protein kinase 4 (STK4)], the mammalian homologue Hippo in *Drosophila*, regulates cell proliferation, apoptosis (14) and chromatin condensation (15). Mst1 has been demonstrated to induce cardiomyocyte apoptosis and cardiac dysfunction during myocardial infarction (16, 17), contributing to the progression of atherosclerosis (18) and worsening angiotensin II-mediated cardiac injury (19). The results of our previous studies showed that Mst1 contributes to the development of DCM by regulating apoptosis and autophagy (20, 21), suppressing glucose metabolism (21) and impairing mitophagy (22). Thus, Mst1 plays an important role in the progression of DCM. 

Physical exercise not only is effective for glycaemic control in diabetes (2, 23, 24) but also protects against DCM by suppressing pathological hypertrophy (25), fibrosis (25), and apoptosis (26) and by reducing cardiovascular risks in the diabetic heart (27), although the mechanisms underlying the exercise-mediated cardioprotective effects in DCM remain to be fully elucidated.

Therefore, exercise and Mst1 have the opposite effects on DCM, but the relationship between them is unclear. Contracting muscle-derived miRNAs (28, 29) may be responsible for the regulation of Mst1 by exercise, leading to exercise-induced cardiac protection in DCM. In the present study, we observed that exercise-mediated miR-486a-5p up-regulation correlated with the inhibition of Mst1 and protected against DCM. Our results likely shed new light on the precise control of DCM. 

## Materials and Methods

***Animals***

Six- to 8-week-old C57BL/6 male mice were obtained from the Experimental Animal Centre of the Air Force Medical University (Xi’an, China), and Mst1-transgenic (Tg-Mst1) mice in a C57BL/6 background were purchased from K&D Gene Technology (Wuhan, China). All mice were housed and monitored in the Experimental Animal Centre of the Air Force Medical University in a controlled environment at 24 **°**C with 45% humidity and a 12-hr light/dark cycle. The C57BL/6 mice were randomly allocated into 4 groups of 5 mice each to investigate exercise-induced cardiac protection in mice with diabetes as follows: (a) sedentary C57BL/6 mice (Sed), (b) exercised C57BL/6 mice (ExT), (c) C57BL/6 mice with diabetes (DM), and (d) diabetic C57BL/6 mice with exercise intervention (DM+ExT). The Tg-Mst1 and control mice were randomly allocated to the following groups (n=5 each) to test whether Mst1 functions as a regulator of exercise-related protective effects: (a) Sed, (b) DM, (c) DM+ExT, (d) sedentary Mst1-transgenic mice (Tg-Mst1), (e) diabetic Tg-Mst1 mice (Tg-Mst1+DM), and (f) diabetic Tg-Mst1 mice with exercise intervention (Tg-Mst1+DM+ExT). Subsequently, the mice underwent exercise training and streptozotocin (STZ) treatment as described in the following sections. At the endpoint of the experiments, the mice were sacrificed via 10% CO_2_ inhalation and the hearts were collected for further experiments. The experimental procedures and animal care were performed in accordance with to the Guide for the Care and Use of Laboratory Animals (30) and approved by the Laboratory Animal Welfare and Ethics Committee of the Air Force Medical University (No. IACUC-20180113).

***Establishment of diabetic mouse models***

A 5-day low-dose STZ protocol was used to obtain a higher success rate of modelling, lower toxicity (31), and greater resemblance to the clinical presentation (32). STZ was administrated by intraperitoneal injection to induce diabetes in C57BL/6 or Tg-Mst1 mice (Figure S1). STZ (Cat.: S0130; Sigma-Aldrich, St. Louis, MO, USA) was dissolved in 0.1 mol/l citrate buffer (pH 4.5) and then injected intraperitoneally into mice at a dose of 50 mg/kg body weight per day for 5 consecutive days. One week after intraperitoneally administering the last dose of STZ, the glucose levels of the mice were detected using a Yuwell 580 glucometer (Jiangsu YUYUE Medical Equipment & Supply Co., Ltd, Nanjing, China), and the mice with a blood glucose level over 16.6 mmol/l were considered diabetic and maintained for future experiments.

***Exercise protocol***

The exercise groups (ExT, DM+ExT, and Tg-Mst1+DM+ExT) were subjected to treadmill exercise (BW-ZH-PT Animal Treadmill; Shanghai BioWill Co., Shanghai, China) using a modified protocol from a previous study (33). Briefly (Figure S1), the training programme consisted of adaptive and regular phases. After confirmation of the well-established diabetes model, the mice were adapted to run on the treadmill for 30 min once per day over 5 days at gradually increased speeds (8, 11, 15, 18, and 22 cm/sec, 4° slope, each step for 6 min). Subsequently, the animals were exercised regularly at a speed of 22 cm/sec for 60 mins per day, 5 days a week for 12 weeks. The basic parameters of each mouse with or without exercise training were determined and are presented in Table SI. 

***Cardiac function evaluation***

Transthoracic M-mode echocardiography was performed to evaluate cardiac function using a high-resolution ultrasound imaging system (Vevo 2100; VisualSonics, Toronto, Ontario, Canada) as previously described (34). Following 2% isoflurane inhalation anaesthesia and the depilation of mice, the left ventricular end-systolic internal dimension (LVESD) and left ventricular end-diastolic internal dimension (LVEDD) of each mouse was measured during the systolic and diastolic phases respectively, while the left ventricular ejection fraction (LVEF) and left ventricular fractional shortening (LVFS) were calculated via computed algorithms. 

***Cardiac fibrosis assessment***

Cardiac fibrosis was evaluated via Masson’s trichrome staining and immunoblotting. A Masson’s Trichrome Stain kit (Cat.: G1340; Solarbio, Beijing, China) was used for Masson’s trichrome staining according to the manufacturer’s instructions. The details of the Western blot analysis of fibrotic proteins are described in the *Western blot assay* section. 

For collagen staining with Masson’s trichrome, fresh cardiac tissues were prepared as 3-μm sections after being fixed (4% paraformaldehyde at 25 °C for 24 hr), dehydrated (70, 80, 90, 95 and 100% ethanol for 1 hr each, 25 °C), cleared (xylene, 2 changes for 1 hr each, 25 °C), and paraffin embedded (paraffin wax, 2 changes, 2 hr each, 52-56 °C; solidification on frozen plates afterwards). Following dewaxing (xylene, 2 changes for 30 min each, 25 °C) and rehydration (100, 95, 90, 80 and 70% ethanol for 5 min, 25°C), the paraffin-embedded tissue sections were stained with Weigert’s iron haematoxylin solution for 5 min. After differentiation and bluing, the tissue sections were stained in Biebrich scarlet/acid fuchsin solution for 5 min. The sections were then rinsed in phosphomolybdic/phosphotungstic acid solution followed by staining with an aniline blue solution for 5 min. After dehydration and clearing, the tissue sections were covered by coverslips and were analysed using a fluorescence microscope (DM4000; Leica Microsystems, Wetzlar, Germany). Five random fields from each section were collected, and the degree of fibrosis was quantified as the collagen volume fraction (CVF; the ratio of myocardial collagen area to total vision area) using Image-Pro Plus (Media Cybernetics Inc., Rockville, MD, USA). 

***Apoptosis determination***

Cardiomyocyte apoptosis was determined by TUNEL and Western blotting. The TUNEL assay was performed using an *In Situ* Cell Death Detection kit (Cat.: 11684817910; Roche, Basel, Switzerland) according to the manufacturer’s instructions, and the details of the Western blot analysis of apoptosis markers are described in a later section. 

To assess apoptosis in cardiac tissues, paraffin-embedded cardiac tissue sections were incubated in a Proteinase K working solution for 30 min at 37 °C after being deparaffinized and rehydrated. To investigate apoptosis in primary neonatal mouse cardiomyocytes, cell samples were fixed with 4% paraformaldehyde for 1 hr at 25 **°**C and then permeabilized in 0.1% Triton X-100 for 2 min at 4 **°**C. 

Subsequently, the tissue sections or cells were stained with the TUNEL reaction mixture and incubated for 60 min at 37 **°**C under a humidified atmosphere in the dark. After being rinsed in PBS, the tissue sections or cells were counterstained with DAPI Staining Solution (Cat.: C1005; Beyotime Biotechnology, Shanghai, China). After being embedded with the antifade solution (Cat.: P0126; Beyotime Biotechnology, Shanghai, China), the samples were analysed under a fluorescence microscope (DM4000; Leica Microsystems, Wetzlar, Germany). The numbers of TUNEL-positive cells and total cells were counted within 10 random fields in each sample by a researcher who was not directly involved in the study, and the apoptosis index (TUNEL-positive cells/total cells) was determined to semiquantitatively assess apoptosis. 

***Cultivation of neonatal mouse cardiomyocytes***

Primary neonatal mouse cardiomyocytes were harvested from 1-day-old neonatal C57BL/6 mice according to a standard protocol (35). In brief, mice were sacrificed following 10% CO_2 _inhalation, and the hearts were carefully removed. After being minced into small pieces (0.5-1 mm^3^), the hearts were incubated with 0.25% trypsin (Cat.: C0201; Beyotime Biotechnology, Shanghai, China) at 4 **°**C overnight. The predigested cardiac tissues were then mixed with collagenase II (Cat.: 1148090; Sigma-Aldrich, St. Louis, MO, USA) and incubated at 37 **°**C with gentle agitation for 15 min. Subsequently cardiac cells were obtained following cell straining and centrifugation for 5 min at 50 × g and were plated in 10-cm culture dishes and incubated for 2 hr in an incubator for preplating. After incubating, nonadherent cardiomyocytes were transferred to 6-well culture plates containing complete DMEM (Cat.: SH30022.01; GE Life Sciences, USA) supplemented with 10% foetal bovine serum (Cat.: 900-108; GEMINI Bio-Products, Woodland, CA, USA), and 1% penicillin/streptomycin (Cat.: C0222; Beyotime Biotechnology, Shanghai, China) and were maintained in a humidified incubator at 37 **°**C under an atmosphere with 5% CO_2_. The cardiomyocytes were randomly allocated into the following groups to verify the transfection efficiency and Mst1 protein levels after mimic/inhibitor transfection: (a) negative control (Control), (b) cells with miR-486a-5p mimic transfection (miR mimic), and (c) cells with miR-486a-5p inhibitor transfection (miR inhibitor). To probe the effect of miR-486a-5p on apoptosis *in vitro*, cardiomyocytes were randomly assigned to the following groups: (a) negative control (Control), (b) cardiomyocytes cultured under hyperglycaemic conditions (HG), (c) control cells with miR-486a-5p mimic transfection (Control+miR mimic), (d) HG cultured cells with miR-486a-5p mimic transfection (HG+miR mimic), (e) control cells with miR-486a-5p inhibitor transfection (Control+miR inhibitor), and (f) HG cultured cells with miR-486a-5p inhibitor transfection (HG+miR inhibitor). Furthermore, the relationship between miR-486a-5p and Mst1 in cardiomyocytes was evaluated. Cells were first divided randomly into 2 groups to confirm the transfection of the Mst1 over-expressing adenovirus: (a) negative control (Control) and (b) cells with adenovirus transfection (Ad-Mst1). Then, the cells were randomly allocated into the following groups to detect whether miR-486a-5p protects cardiomyocytes by targeting Mst1: (a) negative control (Control), (b) cardiomyocytes cultured under hyperglycaemic conditions (HG), (c) control cells with miR-486a-5p mimic transfection (Control+miR mimic), (d) HG cultured cells with miR-486a-5p mimic transfection (HG+miR mimic), (e) control cells with miR-486a-5p mimic and adenovirus transfection (Control+miR mimic+Ad-Mst1), and (f) HG cultured cells with miR-486a-5p mimic and adenovirus transfection (HG+ miR mimic+Ad-Mst1). 

***RNA extraction and RT-qPCR***

Total RNA was isolated from cardiac tissues or cells using an RNAprep pure Tissue or Cell kit (Cat.: DP431 or DP430; Tiangen Biotech Co., Beijing, China). To assess the mRNA levels of Mst1, RNA was reverse-transcribed to cDNA using a RevertAid First Strand cDNA Synthesis kit (Cat.: K1622; Thermo Fisher Scientific, Waltham, MA, USA), and RT-qPCR was performed using on an Applied Biosystems StepOnePlus Real-Time PCR system (Thermo Fisher Scientific, Waltham, MA, USA). Each 50 µl reaction contained 25 μl of 2×UltraSYBR Mixture (Cat.: CW0957; CWBIO, Beijing, China), 1 μl of primer (10 μmol/l), 2 μl of cDNA template and 21 μl of ddH_2_O. The thermocycling program consisted of 40 cycles of denaturation for 10 sec at 95 °C, annealing for 30 sec at 60 °C, and extension for 1 min at 72°C. To evaluate the expression of miR-486a-5p, an miRcute Plus miRNA First-Strand cDNA kit (Cat.: KR211; Tiangen Biotech Co., Beijing, China) and an miRcute Plus miRNA qPCR kit (Cat.: FP411; Tiangen Biotech Co., Beijing, China) were used for reverse transcription and RT-qPCR, respectively, following the manufacturer’s instructions. Each 50 μl reaction contained 25 μl of 2×miRcute plus miRNA PreMix, 1 μl of primer (10 μmol/l), 2 μl of miRNA-derived first-strand cDNA, 4 μl of 50× ROX Reference Dye and 17 μl of ddH_2_O. The thermocycling conditions were as follows: 95 °C for 15 min followed by 42 cycles of 94 °C for 20 sec and 60 °C for 34 sec.

The results were normalized to the expression of β-actin (for Mst1) or U6 (for miR-486a-5p), and the 2^-ΔΔCt^ method was used to calculate the relative expression of target genes. The primer sequences were as follows: Mst1, forward (5’-AGCCCTCACGTAGTCAAGTAT-3’) and reverse (5’-TCTTGTTCCGTAGCCGAATGATA-3’); β-actin, forward (5’-GGCTGTATTCCCCTCCATCG-3’) and reverse (5’-CCAGTTGGTAACAATGCCATGT-3’); miR-486-a-5p, forward (5’-GCAGTCCTGTACTGAGCTG-3’) and reverse (5’-GTCCAGTTTTTTTTTTTTTTTCTCG-3’); U6, forward (5’-GCGCGTCGTGAAGCGTTC-3’) and reverse (5’-GTGCAGGGTCCGAGGT-3’). 

***Western blot analysis***

The protein levels of Mst1 and apoptosis markers in cardiac tissue and cell samples were analysed via standard immunoblotting using previously described protocols (20). Briefly, cardiac tissue and cell samples were lysed with RIPA lysis buffer on ice for 1 hr, after which the BCA assay was used for protein quantification. An equal amount of total protein (20 μg per sample) was separated by 12% SDS-PAGE and transferred to PVDF membranes. Then, after blocking with 10% fat-free milk, the membranes were incubated with specific primary antibodies at 4 °C overnight, which was followed by an incubation with an HRP-conjugated secondary antibody at 37 °C for 40 min. Signals were developed using Bio-Rad Clarity Western ECL Substrate (Cat.: 1705061; Bio-Rad Laboratories, Hercules, CA, USA) and visualized with a Bio-Rad ChemiDoc XRS+ imaging system (Bio-Rad Laboratories, Hercules, CA, USA). The greyscale densities of bands were quantified by using ImageJ (Version: 1.52a; National Institutes of Health, Bethesda, MD, USA). Primary antibodies against the following proteins were used: collagen I (rabbit anti-mouse polyclonal; 1:1000; Cat.: ab34710, Abcam, Cambridge, MA, USA), collagen III (rabbit anti-mouse monoclonal; 1:5000; Cat.: ab7778, Abcam, Cambridge, MA, USA), Mst1 (rabbit anti-mouse monoclonal; 1:1000; Cat.: ab232551, Abcam, Cambridge, MA, USA), cleaved-caspase 3 (rabbit anti-mouse polyclonal; 1:1000; Cat.: ab2302, Abcam, Cambridge, MA, USA), cleaved-caspase 9 (rabbit anti-mouse polyclonal; 1:1000; Cat.: #9509S, CST, Danvers, MA, USA), cleaved-PARP (rabbit anti-mouse monoclonal; 1:1000; Cat.: ab32064, Abcam, Cambridge, MA, USA), and GAPDH (rabbit anti-mouse monoclonal; 1:500; Cat.: sc-32233, Santa Cruz, Dallas, Texas, USA). In addition, a goat anti-rabbit polyclonal secondary antibody conjugated to HRP (Cat.: ZB-2301; ZSGB-Bio, Beijing, China) was used. 

***Bioinformatic analyses***

TargetScanMouse 7.2 (www.targetscan.org), miRDB (mirdb.org), and TarBase V.8 (http://carolina.imis.athena-innovation.gr/diana_tools/web/index.php?r=tarbasev8%2Findex/) were used to predict miRNAs that target Mst1 with the following filters: “species: mouse” and “gene symbol: Mst1/STK4”. A Venn diagram was generated to determine the overlap among the database-predicted miRNAs and those induced by exercise (29). 

***Cell transfection***

The miR-486a-5p mimics, miR-486a-5p inhibitor and respective negative controls (GenePharma Co., Shanghai, China) were transfected into cells using Lipofectamine 2000 reagent (Cat.: 11668-019; Thermo Fisher Scientific, Waltham, MA, USA) following the manufacturer’s instructions. In brief, primary neonatal mouse cardiomyocytes were seeded in 6-well plates at a density of 1×10^6^ per well and incubated with complexes containing 5 μl RNA and 5 μl Lipofectamine 2000 Reagent at 37 °C under an atmosphere with 5% CO_2_. The medium was replaced with complete medium 6 h after transfection. 

The Mst1-over-expressing adenovirus was constructed by Hanbio Technology (Shanghai, China). In brief, mouse Mst1 cDNA was PCR amplified [primer sequences: forward (atatgctagcATGGAGACCGTGCAGCTGAGGAACCCA) and reverse (atatcccgggGAAGTTCTGTTGCCTCCTCTTCTT)], restriction enzyme digested, ligated and cloned into the shuttle vector pHBAd-U6-CMV. The shuttle vector over-expressing Mst1 and the backbone vector pBHGlox(delta)E1, 3Cre were transformed into competent cells for amplification. After sequencing and identification, the plasmids were extracted and cotransfected into 293 cells for recombination. The Mst1-over-expressing adenovirus was obtained after viral packaging, collection, amplification, and purification. The adenovirus harbouring Mst1 (Ad-Mst1) and the negative control (MOI:100) were transduced into cardiomyocytes for 6 hr before complete medium replacement. Further experiments were performed within 48 hr. The relevant sequences were as follows: miR-486a-5p mimics, forward (5’-UCCUGUACUGAGCUGCCCCGAG-3’) and reverse (5’-CGGGGCAGCUCAGUACAGGAUU-3’); and miR-486a-5p inhibitor (CUCGGGGCAGCUCAGUACAGGA). 

***Dual-luciferase reporter gene assay***

The length of the mouse Mst1 3’UTR is 3709 bp, and the predicted miR-486a-5p binding sites in the 3’ UTR are located between 1947 and 1953 bp. The region between 1741 and 2160 bp was PCR amplified [primers: forward (5’-TAGACCCCAGGAGCAGAGAC-3’) and reverse (5’-CAGCTTCCGAGGCCAGTG-3’)] and cloned into the dual luciferase miRNA target expression vector pmirGLO (Cat.: E1330, Promega, Madison, WI, USA). After pretreatment with the miR-486a-5p mimics or negative control, cells were transfected with the pmirGLO negative control, pmirGLO-Mst1-3’UTR-wildtype, or pmirGLO-Mst1-3’ UTR-mutant vector using Lipofectamine 2000 reagent. Forty-eight hours after transfection, the cells were analysed for normalized firefly luciferase activity using the Dual-Glo Luciferase Reporter Assay System (Cat.: E2920, Promega, Madison, WI, USA) according to the manufacturer’s protocol. 

***Statistical analyses***

The data are presented as the means±SEM of triplicate experiments, and the analyses were performed using GraphPad Prism 8.0 (GraphPad Software Inc., San Diego, CA, USA). Student’s t-test or ANOVA followed by a *post hoc *test was used to assess group differences, and *P*<0.05 was considered to indicate a significant difference. 

**Figure 1:**
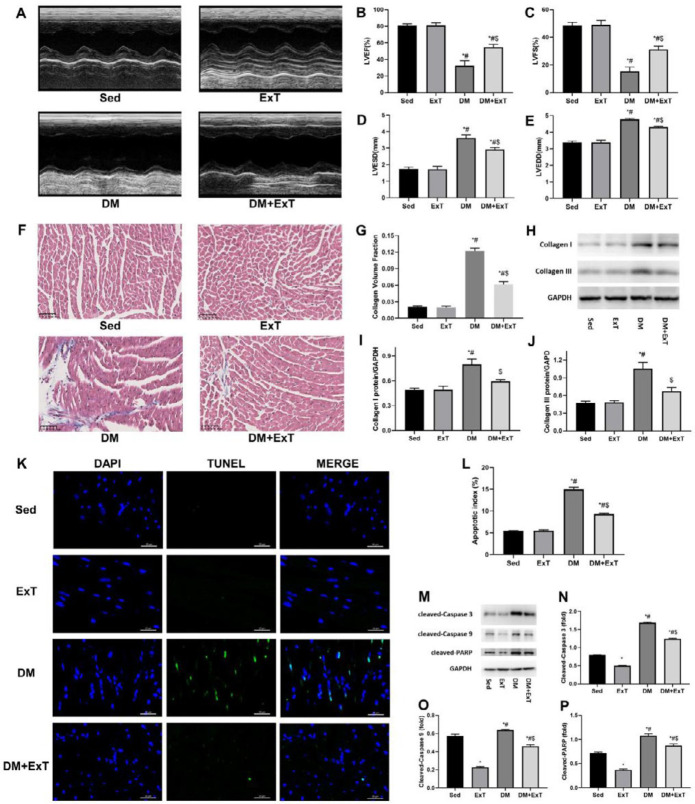
Exercise alleviates cardiac remodelling in mice with DCM. (A) Representative transthoracic M-mode echocardiographs. (B-E) Measurements of LVEF, LVFS, LVESD and LVEDD (n=5). (F) Representative images of Masson’s trichrome staining showing collagen deposition. (G) Quantification of collagen volume fraction using Image-Pro Plus (n=5). (H-J) Representative immunoblots and semiquantitative analysis of collagen I and collagen III. (K) Representative immunofluorescent images of staining with DAPI (blue) and TUNEL (green). (L) Quantitative assessments of apoptotic indexes (n=5). (M-P) Representative immunoblots and semiquantitative analysis of cleaved-caspase 3, cleaved-caspase 9, and cleaved-PARP. Sed, sedentary C57BL/6 mice; ExT, exercised C57BL/6 mice; DM, C57BL/6 mice with diabetes; DM+ExT, diabetic C57BL/6 mice with exercise intervention; LVEF, left ventricular ejection fraction; LVFS, left ventricular fractional shortening; LVESD, left ventricular end-systolic internal dimension; LVEDD, left ventricular end-diastolic internal dimension. The data are presented as the means±SEM of triplicate experiments, and group differences were determined by one-way ANOVA followed by Tukey’s multiple comparisons test. *, *P*<0.05 vs Sed; #, *P*<0.05 vs ExT; $, *P*<0.05 vs DM

**Figure 2:**
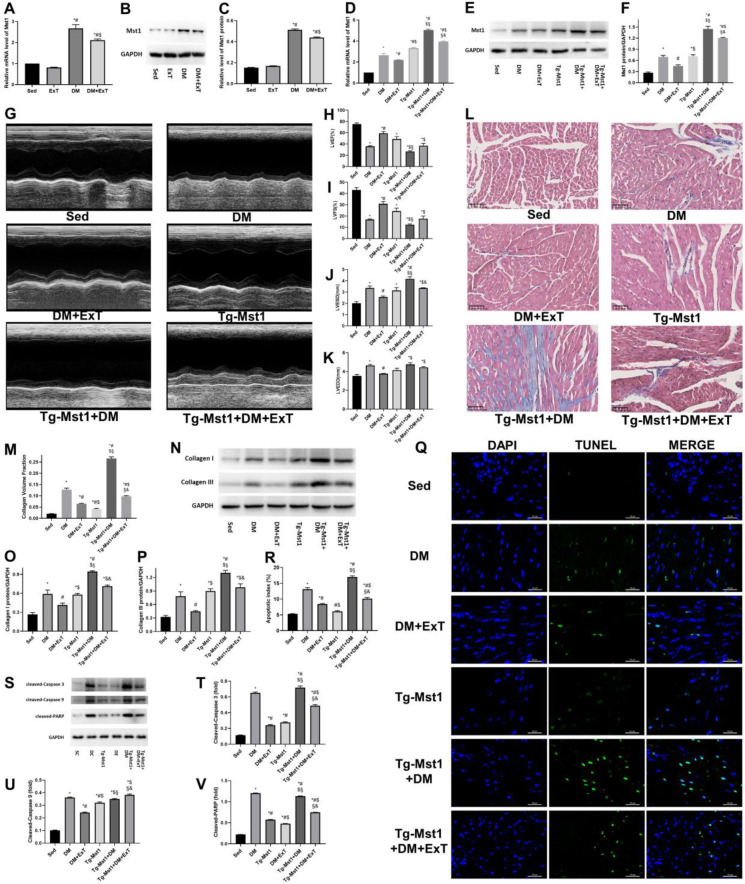
Exercise protects against DCM by downregulating Mst1. (A) Relative Mst1 mRNA levels evaluated by RT-qPCR; *, *P*<0.05 vs Sed; #, *P*<0.05 vs ExT; $, *P*<0.05 vs DM. (B, C) Evaluation of Mst1 protein levels by immunoblotting and the relevant relative greyscale values; *, *P*<0.05 vs Sed; #, *P*<0.05 vs ExT; $, *P*<0.05 vs DM. (D-F) Mst1 expression determination via RT-qPCR and immunoblotting; *, *P*<0.05 vs Sed; #, *P*<0.05 vs DM; $, *P*<0.05 vs DM+ExT; §, *P*<0.05 vs Tg-Mst1; &, *P*<0.05 vs Tg-Mst1+DM. (G-K) Representative echocardiograms and assessments of cardiac function (n=5); *, *P*<0.05 vs Sed; #, *P*<0.05 vs DM; $, *P*<0.05 vs DM+ExT; §, *P*<0.05 vs Tg-Mst1; &, *P*<0.05 vs Tg-Mst1+DM. (L, M) Representative images of Masson’s trichrome staining and respective collagen volume fractions (n=5); *, *P*<0.05 vs Sed; #, *P*<0.05 vs DM; $, *P*<0.05 vs DM+ExT; §, *P*<0.05 vs Tg-Mst1; &, *P*<0.05 vs Tg-Mst1+DM. (N-P) Representative immunoblots and semiquantitative analysis of collagen I and collagen III; *, *P*<0.05 vs Sed; #, *P*<0.05 vs DM; $, *P*<0.05 vs DM+ExT; §, *P*<0.05 vs Tg-Mst1; &, *P*<0.05 vs Tg-Mst1+DM. (Q, R) Representative TUNEL staining and corresponding quantification of apoptotic cells (n=5); *, *P*<0.05 vs Sed; #, *P*<0.05 vs DM; $, *P*<0.05 vs DM+ExT; §, *P*<0.05 vs Tg-Mst1; &, *P*<0.05 vs Tg-Mst1+DM. (S-V) Representative blots and semiquantitative analyses of apoptotic markers; *, *P*<0.05 vs Sed; #, *P*<0.05 vs DM; $, *P*<0.05 vs DM+ExT; §, *P*<0.05 vs Tg-Mst1; &, *P*<0.05 vs Tg-Mst1+DM. Sed, sedentary C57BL/6 mice; DM, C57BL/6 mice with diabetes; DM+ExT, diabetic C57BL/6 mice with exercise intervention; Tg-Mst1, sedentary Mst1-transgenic mice; Tg-Mst1+DM, diabetic Tg-Mst1 mice; Tg-Mst1+DM+ExT, diabetic Tg-Mst1 mice with exercise intervention. The data are presented as the means±SEM of triplicate experiments, and comparisons among groups were assessed by one-way ANOVA and Tukey’s multiple comparisons test

**Figure 3:**
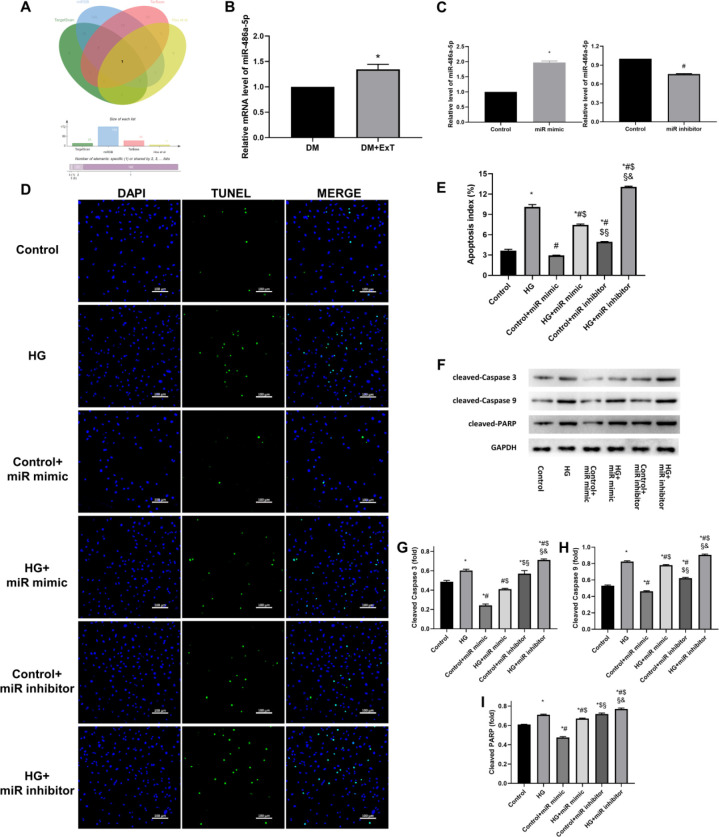
Exercise-induced enhancement of miR-486a-5p and potentially downregulation of Mst1 inhibited apoptosis in cardiomyocytes. (A) Bioinformatic analysis based on 3 public databases predicted that miR-486a-5p expression is enhanced by exercise (29) and can target Mst1. (B) Relative miR-486a-5p levels assessed by RT-qPCR; *, *P*<0.05 vs DM. (C) miR-486a-5p levels after miR-486a-5p mimic or inhibitor transfection, as assessed by RT-qPCR; *, # *P*<0.05 vs Control. (D) Representative immunofluorescence images of staining with DAPI (blue) and TUNEL (green). (E) Quantitative assessments of apoptotic indexes; *, *P*<0.05 vs Control; #, *P*<0.05 vs HG; $, *P*<0.05 vs Control+miR mimic; §, *P*<0.05 vs HG+miR mimic; &, *P*<0.05 vs Control+miR inhibitor. (F-I) Representative immunoblots and semiquantitative analysis of biomarkers of apoptosis; *, *P*<0.05 vs Control; #, *P*<0.05 vs HG; $, *P*<0.05 vs Control+miR mimic; §, *P*<0.05 vs HG+miR mimic; &, *P*<0.05 vs Control+miR inhibitor. Control, negative control; miR mimic, cardiomyocytes with miR-486a-5p mimic transfection; miR inhibitor, cardiomyocytes with miR-486a-5p inhibitor transfection; HG, cardiomyocytes cultured under hyperglycaemic conditions; Control+miR mimic, control cells with miR-486a-5p mimic transfection; HG+miR mimic, HG cultured cells with miR-486a-5p mimic transfection; Control+miR inhibitor, control cells with miR-486a-5p inhibitor transfection; HG+miR inhibitor, HG cultured cells with miR-486a-5p inhibitor transfection. The data are presented as the means±SEM of triplicate experiments, and Student’s t-test or ANOVA was used to assess group differences

**Figure 4:**
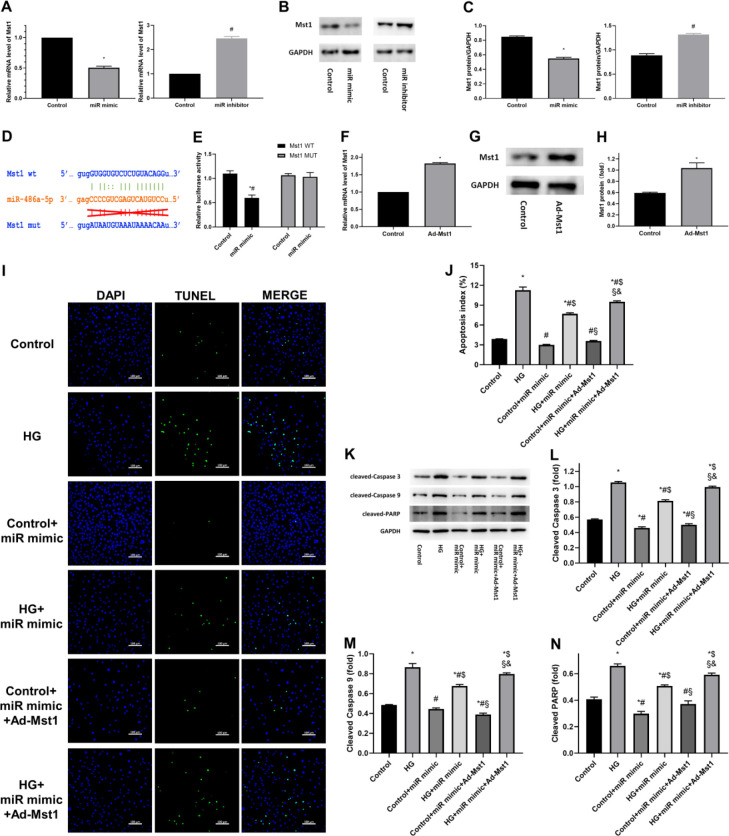
Exercise-induced miR-486a-5p expression suppresses apoptosis by targeting Mst1. (A) Relative Mst1 mRNA levels after transfection of the miR-486a-5p mimic or inhibitor, as assessed by RT-qPCR; *, #, *P*<0.05 vs Control. (B, C) Western blot and semiquantitative analyses of Mst1 expression; *, #, *P*<0.05 vs Control. (D) Predicted target of miR-486a-5p in the Mst1 3’ UTR and relevant mutations. (E) Quantification of relative luciferase activities; *, *P*<0.05 vs Mst1 wildtype control groups; #, *P*<0.05 vs Mst1 mutant groups with miR-486a-5p mimic transfection. (F-H) Mst1 upregulation in cardiomyocytes after Ad-Mst1 transfection; *, *P*<0.05 vs Control. (I, J) Representative TUNEL staining and quantitative analysis of apoptotic cells; *, *P*<0.05 vs Control; #, *P*<0.05 vs HG; $, *P*<0.05 vs Control+miR mimic; §, *P*<0.05 vs HG+miR mimic; &, *P*<0.05 vs Control+miR mimic+Ad-Mst1. (K-N) Representative immunoblots and quantification of apoptotic markers; *, p<0.05 vs Control; #, *P*<0.05 vs HG; $, *P*<0.05 vs Control+miR mimic; §, *P*<0.05 vs HG+miR mimic; &, *P*<0.05 vs Control+miR mimic+Ad-Mst1. Control, negative control; miR mimic, cardiomyocytes with miR-486a-5p mimic transfection; miR inhibitor, cardiomyocytes with miR-486a-5p inhibitor transfection; Ad-Mst1, cardiomyocytes overexpressing Mst1 adenovirus transfection; HG, cardiomyocytes cultured under hyperglycaemic conditions; Control+miR mimic, control cells with miR-486a-5p mimic transfection; HG+miR mimic, HG cultured cells with miR-486a-5p mimic transfection; Control+miR mimic+Ad-Mst1, control cells with miR-486a-5p mimic and adenovirus transfection; HG+miR mimic+Ad-Mst1, HG cultured cells with miR-486a-5p mimic and adenovirus transfection. The data are presented as the means±SEM of triplicate experiments, and Student’s t-test or ANOVA was used to assess group differences

## Results

***Exercise alleviates cardiac remodelling in mice with DCM***

Diabetes was induced in C57BL/6 or Tg-Mst1 mice via an intraperitoneal injection of STZ (Figure S1). M-mode echocardiograms showed that mice in the DM group presented worse cardiac functions than those in the Sed group (Sed) (Figure 1A-E), with the LVEF decreasing from 80.72 to 32.41%, the LVFS decreasing from 48.44 to 15.32%, the LVESD increasing from 1.74 to 3.62 mm, and the LVEDD from 3.38 to 4.77 mm. Masson’s trichrome staining (Figure 1F, G) and collagen I and III immunoblotting (Figure 1H-J) revealed that collagen deposition was more common in the DM group than in the Sed group. In addition, mice in the DM group exhibited more apoptotic cells than the Sed group as determined via TUNEL assay (Figure 1K, L) and Western blot analysis (Figure 1M-P). The above mentioned pathophysiological changes indicated the generation of well-established DCM models. 

A moderate-intensity exercise protocol was used to evaluate the effect of exercise on DCM in mice (Figure S1). Compared to the mice in the DM control group, the exercised diabetic mice (DM+ExT) had significantly higher LVEF and LVFS as well as lower LVESD and LVEDD, demonstrating the better cardiac function of the mice in the DM+ExT group (Figure 1A-E). In addition, the mice in the DM+ExT group showed significantly lower collagen deposition than those in the DM group (Figure 1F-J). Moreover, fewer TUNEL-positive cells were observed in the DM+ExT group than in the DM group, and cleaved-caspase 3, cleaved-caspase 9 and cleaved-PARP levels consistently exhibited a trend towards down-regulation (Figure 1K-P), showing that exercise could inhibit cardiomyocytes apoptosis. Taken together, these results confirmed the cardioprotective effects of exercise, and that exercise rehabilitated the dysfunction of diabetic hearts, attenuated collagen deposition, and alleviated the apoptosis of cardiomyocytes in DCM mice. 

***Exercise protects against DCM by down-regulating Mst1***

To determine whether exercise correlates with Mst1 in DCM, we performed RT-qPCR and immunoblotting analyses to compare the differences in Mst1 expression among the groups. The results (Figure 2A-C) showed that Mst1 mRNA and protein levels were elevated in the DCM mouse models. Furthermore, decreased Mst1 expression (Figure 2A-C) was observed in the mice in the DM+ExT group compared to the DM control mice. As Mst1 aggravates DCM via several of the pathways mentioned above, exercise-induced cardioprotective effects in DCM may require the inhibition of Mst1 function. 

Tg-Mst1 mice were then used to further elucidate whether exercise suppresses Mst1 in DCM. Tg-Mst1 diabetic mouse models were constructed and underwent exercise training similar to that described above (Figure S1), and Mst1 expression was also evaluated in the wildtype and Tg-Mst1 mice (Figure 2D-F). M-mode echocardiograms (Figure 2G-K) and fibrosis assessments (Figure 2L-P) revealed that the Tg-Mst1 diabetic mice (Tg-Mst1+DM+ExT) exhibited worse cardiac function and greater collagen deposition than their diabetic wildtype counterparts (DM+ExT) after regular exercise for 3 months. Moreover, the ratios of apoptotic cardiomyocytes (Figure 2Q, R) and apoptotic markers (Figure 2S-V) were increased in the Tg-Mst1+DM+ExT group compared to the DM+ExT group. These data revealed that Mst1 over-expression during DCM can counteract the cardioprotective effect of exercise, indicating that exercise protects against DCM by down-regulating Mst1. 

***Exercise-induced enhancement of miR-486a-5p and potentially down-regulating of Mst1 inhibited apoptosis in cardiomyocytes***

Next, the connection between exercise and Mst1 was evaluated. Previous studies (28, 29, 36) have shown that miRNAs contribute to exercise-mediated cardiac protection, and in the present study, analysis of the intersection of these miRNAs and those that potentially target the Mst1 gene (Figure 3A) showed that miR-486a-5p may function as a link between exercise and Mst1 in mice. 

The RT-qPCR results confirmed that miR-486a-5p was upregulated in the diabetic hearts of mice after exercise (DM+ExT; Figure 3B). In addition, miR-486a-5p mimics significantly attenuated apoptosis, whereas a miR-486a-5p inhibitor exacerbated apoptosis in cardiomyocytes under hyperglycaemic conditions (Figure 3D, E). These results were consistent with the observed changes in expression of apoptotic biomarkers after miRNA-486a-5p mimic or inhibitor transfection (Figure 3F-I). Thus, exercise may combat DCM by upregulating miR-486-5p, which results in the inhibition of cardiomyocyte apoptosis. 

***Exercise-induced miR-486a-5p expression has an anti-apoptotic role by suppressing Mst1 in cardiomyocytes***

Subsequently, we further assessed whether miR-486a-5p can inhibit Mst1 in cardiomyocytes. Mst1 expression was significantly decreased after miR-486a-5p mimic treatment and was increased after miR-486a-5p inhibitor treatment (Figure 4A-C). Furthermore, a dual-luciferase reporter gene assay was performed to confirm the direct inhibition of Mst1 by miR-486a-5p (Figure 4D, E). The relative luciferase activity in cells harbouring the wildtype Mst1 3’UTR reporter and previously transfected with miR-486a-5p mimics was significantly reduced compared with that in the vector control cells, whereas transfection with miR-486a-5p mimics did not affect the luciferase reporter activity of the mutant Mst1 3’UTR construct. Taken together, these results showed that Mst1 is a target gene of miR-486a-5p and that the enhancement of miR-486a-5p expression by exercise decreased the expression of Mst1 by directly binding to its 3’UTR in cardiomyocytes. 

To verify that miR-486a-5p could repress Mst1 expression, we restored the expression of Mst1 in cardiomyocytes via adenovirus transfection (Figure 4F-H) and observed the effect of Mst1 up-regulation on cardiomyocyte apoptosis. The results showed that the restoration of Mst1 expression could reverse the inhibition of apoptosis mediated by miR-486a-5p mimic transfection (Figure 4I-N). Thus, the above results confirmed that miR-486a-5p has anti-apoptotic effects by suppressing Mst1 expression in cardiomyocytes.

## Discussion

The results of the present study demonstrated that moderate-intensity exercise has cardioprotective effects in DCM model mice by regulating Mst1 expression and that exercise-induced miR-486-5p expression is a potential cardioprotective factor that directly inhibits of Mst1. These findings increase our understanding of the benefits of exercise on cardiovascular diseases, especially DCM, and provide a feasible target to alleviate DCM. 

Our results suggested that Mst1 may be a key molecule mediating the cardioprotective effects of exercise on DCM. Among the mechanisms involved in the pathogenesis of DCM reviewed elsewhere (37), cardiomyocyte death is one of the most important molecular foundations of the progression of DCM. In line with studies performed in other labs, our previous studies (20, 21) also revealed that Mst1 can promote cardiomyocyte apoptosis in DCM. As the inhibition of apoptosis is one of the primary mechanisms involved in the favourable effects mediated by exercise on the heart, we hypothesized that exercise may protect against DCM by suppressing Mst1. The results of the present study confirmed that Mst1 expression was down-regulated after exercise and Mst1 over-expression could reduce the cardiac protection conferred by exercise in DCM. Therefore, exercise can inhibit cardiomyocyte apoptosis by down-regulating Mst1 in DCM, ameliorating cardiac dysfunction and protecting against DCM.

We also observed that exercise-induced miR-486a-5p expression could suppress the apoptosis of cardiomyocytes and that miR-486a-5p could directly inhibit Mst1 in DCM. These data are consistent with those of previous studies (28, 29) in which dynamic changes in the levels of miRNAs were observed in response to exercise, and several miRNAs can inhibit a variety of pathophysiological state-induced forms of cardiomyocyte apoptosis, thereby protecting the heart from injuries. In addition, our results indicated that miR-486-5p can inhibit the apoptosis of cardiomyocytes under high glucose conditions, with concomitant down-regulation of Mst1 expression. These results are in accordance with the idea that miR-486a-5p can suppress apoptosis by influencing other growth-related genes, such as PTEN (38) and FoxO1 (39). The relationship between exercise intensity and cardioprotection should exhibit a parabolic-like effect, as evidenced by the results of a study by Chekroud Chekroud *et al*. who showed that exercise can reduce all-cause mortality, whereas a single-bout duration of more than 60 min may have adverse effects (40). In addition, even though some studies have shown that moderate- to high-intensity physical exercise may decrease the burden of non-communicable diseases, exercise tolerance can be impaired due to underlying diseases, making high intensity physical fitness less suitable and beneficial for populations with multiple comorbidities. Thus, a moderate-intensity exercise protocol could aid in preventing the development or delay the progression of cardiomyopathy in patients with DM, and the use of exogenous miR-486a-5p may be beneficial for patients with DCM who are intolerant to physical activities. However, this type of novel miRNA-based therapy requires further investigation. 

There were several limitations to our present study. As we used the STZ-induced type 1 diabetes model in the present study, the results may not be representative of all types of diabetes. Since db/db and ob/ob mice are infertile, genetically engineered mouse models with simultaneous mutations in Mst1 and the leptin receptor are poorly accessible, while STZ-induced DCM models are widely used. Therefore, we believe that the model used in the present study is relatively feasible. Moreover, we focused on cardiac dysfunction related to diabetes, namely, hyperglycaemic damage to cardiomyocytes. The use of STZ to increase the blood glucose level of animals is a reasonable approach, and the results of such experiments were also consistent with those obtained using cells cultured under hyperglycaemic conditions. However, our results were primarily obtained from studies of mice, the heart characteristics of which are different from those of humans. In addition, some diagnostic criteria of human-like echocardiographic parameters should not be applied to mice and other species. Therefore, we used an approach that is highly specific for small animal research (41) to reduce the impact of species differences in the present study, and the results will be verified in well-designed clinical trials in the future. 

## Conclusion

The results of the present study suggest that regular exercise can alleviate cardiac remodelling during DCM by enhancing the expression of miR-486a-5p, which directly suppresses the function of the key apoptotic regulator Mst1, thereby playing a crucial role in protecting the heart. Our findings may provide a better understanding of the protective effects of exercise on the heart, and suggest a novel strategy to treat DCM.
